# Nutritional and Antioxidant Profile of Brown *Eragrostis tef* (Zucc.) Trotter Flour in Blends with *Glycine max* (L.) Merr. Flour

**DOI:** 10.3390/molecules31020365

**Published:** 2026-01-20

**Authors:** Shewangzaw Addisu Mekuria, Kamil Czwartkowski, Joanna Harasym

**Affiliations:** 1 Department of Biotechnology and Food Analysis, Wroclaw University of Economics and Business, Komandorska 118/120, 53-345 Wroclaw, Poland; shewangzaw.mekuria@ue.wroc.pl; 2 College of Veterinary Medicine and Animal Sciences, University of Gondar, Gondar P.O. Box 196, Ethiopia; 3 Department of Agroengineering and Quality Analysis, Wroclaw University of Economics and Business, Komandorska 118/120, 53-345 Wroclaw, Poland; kamil.czwartkowski@ue.wroc.pl; 4 Adaptive Food Systems Accelerator-Science Centre, Wroclaw University of Economics and Business, 53-345 Wroclaw, Poland

**Keywords:** composite flour, antioxidant capacity, pasting properties, protein-starch interactions, gluten-free, amino acid complementation

## Abstract

The still-growing demand for nutritious gluten-free products necessitates the development of a composite flour that addresses the nutritional deficiencies common in conventional gluten-free formulations. This study aimed to comprehensively characterize brown teff (*Eragrostis tef* (Zucc.) Trotter) and soybean (*Glycine max* (L.) Merr.) composite flours at 0%, 10%, 20%, 30%, and 40% soybean inclusion levels (*w*/*w*) to establish evidence-based formulation guidelines for future products. Proximate composition, antioxidant properties (total polyphenol content—TPC, antioxidant capacity vs. 2,2-diphenyl-1-picrylhydrazyl radical—DPPH and 2,2′-azino-bis(3-ethylbenzothiazoline-6-sulfonic acid radical—ABTS, ferric reducing antioxidant power—FRAP), particle size distribution, pasting properties, color characteristics, and molecular fingerprints (Fourier transform infrared spectroscopy—FTIR) were evaluated. A principal component analysis (PCA) was employed to identify compositional–functional relationships. Soybean inclusion significantly enhanced protein content from 9.93% (pure teff) to 23.07% (60:40 blend, dry matter), fat from 2.14% to 10.47%, and fiber from 3.43% to 6.72%. The antioxidant capacity increased proportionally with soybean content, with a 40% inclusion yielding FRAP values of 5.19 mg FeSO_4_/g DM and TPC of 3.44 mg GAE/g DM. However, pasting viscosity decreased notably from 12,198.00 mPa·s (pure teff) to 129.00 mPa·s (60:40 blend), indicating a reduced gel-forming capacity caused by soybean addition. PCA revealed that nutritional composition (PC1: 70.6% variance) and pasting properties (PC2: 21.0% variance) vary independently, suggesting non-additive functional behavior in blends. Brown teff–soybean blends at a 20–30% soybean inclusion optimize the balance between protein enhancement, antioxidant preservation, and the maintenance of functional properties suitable for traditional applications, providing a nutritionally superior alternative for gluten-free product development.

## 1. Introduction

The global demand for gluten-free diets has increased significantly due to celiac disease (affecting approximately 1% of the population), non-celiac gluten sensitivity (ranging from 0.5% to 13%), and consumer health preferences [1,2,3,4]. The market is projected to exceed USD 7.5 billion by 2027 [5,6]. However, conventional gluten-free products often suffer from inferior nutritional value, with lower protein, fiber, and micronutrient contents compared to gluten-containing counterparts [7,8]. Among naturally gluten-free alternatives, teff (*Eragrostis tef* (Zucc.) Trot.), an Ethiopian C4 grass, has emerged as a promising candidate due to its exceptional nutritional profile and climate resilience [9,10].

Brown (red) teff varieties are economically accessible while offering superior functional properties compared to white teff [11,12]. These pigmented varieties exhibit elevated bioactive compounds, with a total polyphenolic content of 118.6–196.7 mg GAE/100 g (free) and 141.1–195.1 mg GAE/100 g (bound), and an antioxidant capacity of 153.8–187.0 mg AAE/100 g, as determined by the DPPH assay [13]. Substantial bound polyphenolics may survive upper gastrointestinal passage, providing a sustained antioxidant activity [13]. Brown-seeded accessions constitute approximately 45% of germplasm collections with higher seed yields [9]. Teff exhibits an atypical cereal profile that addresses key nutritional deficiencies commonly observed in gluten-free products, specifically the low protein content, reduced dietary fiber, and diminished micronutrient density characteristic of refined flour-based formulations. It contains 3.7 g/16 g N lysine—substantially higher than durum wheat (2.1 g/16 g N)—and is rich in leucine, isoleucine, and valine [14,15]. Obligatory whole-grain consumption (due to the approximately 1 mm seed size) ensures a high fiber content (2.6–3.8%) and exceptional mineral density [16], directly counteracting the inadequacies of refined flour-based gluten-free products [7,17]. However, teff’s primary limitation is a deficiency in sulfur-containing amino acids (SAAs)—methionine and cysteine—the limiting factor for optimal protein utilization per WHO/FAO patterns [18,19,20].

Amino acid complementation provides a rationale for blending cereals with legumes to achieve complete amino acid profiles [18,20]. Soybean (*Glycine max* (L.) Merr.); (*Glycine max*), with a Protein Digestibility-Corrected Amino Acid Score (PDCAAS) reaching 1.00 [21], offers an ideal complementation to teff. Soybeans are rich in SAAs, the precise nutrients limiting teff’s protein quality [18]. Blending teff with soybean compensates for SAA deficit while amplifying the total protein and nutritional density [18,19], representing a superior nutritional fortification compared to refined starches and hydrocolloids [22]. Previous studies identified effective ratios of 60–70% teff with 20–30% soybean flour, achieving protein contents up to 17.92% and in vitro protein digestibility of 87.62% [23,24].

Despite these foundations, critical knowledge gaps persist. While research has explored proximate composition and protein enhancement for specific applications [23,24], the comprehensive characterization of nutritional, antioxidant, and physicochemical properties across varying blend ratios under different solvent extraction systems remains limited [25,26]. How incremental soybean addition affects brown teff’s high polyphenolic content—and how increasing teff content impacts soybean’s bioactive isoflavones—remains unanswered. Traditional applications (injera, porridges) require specific rheological properties (liquid consistency) that may be compromised by the excessive inclusion of soybeans, establishing practical formulation limits that are not systematically investigated. Understanding compositional–functional relationships across the lower and limited formulation space (0–40% soybean) is essential for evidence-based product development [2,8].

This study aims to comprehensively characterize the nutritional, antioxidant, physicochemical, and rheological properties of gluten-free composite flours from brown tef and soybean across a 0–40% soybean inclusion. The specific objectives are (1) to evaluate the proximate composition and heavy metal content across blend ratios; (2) to assess the antioxidant capacity via multiple assays (total phenolic content, FRAP, DPPH, ABTS, reducing sugars) to characterize the trade-offs between protein fortification and antioxidant preservation; (3) to characterize particle size and color properties; (4) to determine molecular fingerprints via FT-IR and compositional patterns through principal component analysis; and (5) to evaluate pasting behavior and viscosity to establish functional limitations for paste-forming applications including injera and porridge.

This research will establish evidence-based guidelines for optimizing brown teff–soybean composite flours, balancing protein enhancement, antioxidant preservation, and rheological functionality for nutritionally superior gluten-free products.

## 2. Results and Discussion

### 2.1. Proximate Composition and Heavy Metal Content

The proximate composition of the flours and their blends, including ash, protein, fat, fiber, carbohydrate (CoH), heavy metals, and energy content, is presented in Table 1.

The level of ash content, reflecting the mineral composition of the flour blends, increased with a higher soybean content. Pure soybean flour had the highest ash content (4.38%), whereas the 10% soybean inclusion with teff had the lowest value (2.72%). This is consistent with the higher mineral concentration in legumes compared to cereals. The results of the blended flour showed that, as the level of leguminous soybean inclusion increases, the reflecting mineral density rises from 2.72% to 3.27%. Similarly, the soybean content significantly increases protein levels, from 9.93% in teff alone to 42.77% in the soybean blend, demonstrating that soybeans contribute to a higher protein density. This is consistent with soybeans’ known high protein content [27] and shows that blending cereal–legume flours can effectively increase protein content [28,29,30].

Fat and fiber contents also improved as the level of inclusion of soybeans increased, reaching 22.95% and 11.65% in pure soybean flour, compared to 2.14% and 3.43% in pure teff. The higher fat content in soybeans would be due to the higher unsaturated fat contribution of soybeans, while dietary fiber enhances the functional properties of the blends. The fat and fiber contents increased progressively with soybean inclusion, reaching 22.95% and 11.65% in pure soybean compared to 2.14% and 3.43% in teff. Soybeans’ elevated fat reflects their polyunsaturated fatty acid profile, predominantly linoleic and linolenic acids (~60% of total lipids), which increase oxidative vulnerability and hinder water accessibility to starch. The fiber increment represents compositionally distinct fractions: soybean contributes predominantly soluble fiber, enhancing water-binding capacity and emulsification properties, while teff provides primarily insoluble fiber, improving bulk density and textural characteristics. This complementary fiber composition in blends influences functionality—soluble fractions compete with starch for water during hydration, partially explaining the observed viscosity reductions, while insoluble components maintain structural integrity in final products.

Although both soybean and teff contribute fiber, legumes are especially abundant in soluble and insoluble fractions, which improve the blend’s functional qualities. As a result of the starch-rich teff mixing with the protein- and fat-rich soybean, the carbohydrate content dropped from 75.60 ± 0.32 in the 90T10S sample to 56.47 ± 0.32 in the 60T20S sample. The reduction in the carbohydrate concentration in composite flour is mostly due to the dilution effect of soybeans.

The value of energy elevated from 383.32 kcal (100% teff) to 450.62 kcal (100% soybean), with blended samples showing intermediate values (393–412 kcal). This is attributed to the higher protein and fat contributions from soybeans [27], both of which have a higher energy density than carbohydrates.

The heavy metal analysis confirmed food safety compliance for all samples. Cadmium levels (<0.004 mg/kg) and lead concentrations (0.022–0.105 mg/kg) remained well below European Commission Regulation (EC) No. 1881/2006 [31] maximum limits for cereals (Cd: 0.10 mg/kg; Pb: 0.20 mg/kg). Arsenic (<0.010 mg/kg) and mercury (<0.001 mg/kg) were below detection limits.

**Figure 1:**
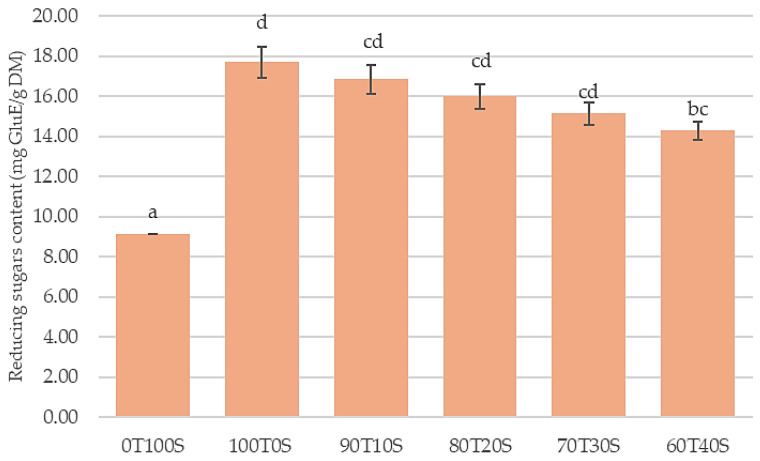
Reducing sugar content of teff and soybean flours and their blends. GluE—glucose equivalents; DM—dry matter. Different lowercase letters indicate significant differences (*p* = 0.05) between the samples.

Reducing the presence of sugars in flour can be native or a result of infestation or previous treatment. Reducing the sugar content reflects the readily hydrolyzable carbohydrate fraction (Figure 1).

Pure teff exhibited the highest reducing sugar content (17.70 mg GluE/g DM), reflecting its starch-rich composition (81.07% carbohydrates). During DNS assay conditions, teff’s readily digestible starch fractions undergo rapid enzymatic hydrolysis, releasing glucose monomers detected as reducing sugars. Progressive soybean inclusion systematically decreased the reducing sugar values—from 16.73 (90T10S) to 14.28 mg GluE/g DM (60T40S)—proportional to the declining total carbohydrate content (75.59–56.48%). Pure soybeans displayed minimal reducing sugars (9.12 mg GluE/g DM) due to their negligible starch content (18.25% carbohydrates). While teff contains resistant starch (~20–40% of total starch), contributing to its low glycemic properties, the majority remains non-resistant amylopectin, susceptible to in vitro enzymatic breakdown. This inverse relationship between soybean inclusion and reducing sugar release confirms starch availability as the primary determinant of enzymatic hydrolysis potential in these composite systems.

It should be noted that the soybean flour underwent thermal processing (boiling for 30 min) to inactivate trypsin inhibitors, which may have partially gelatinized starch and increased reducing sugar accessibility compared to untreated teff. However, the systematic decrease in reducing sugars with increasing soybean inclusion primarily reflects the substantially lower total carbohydrate content of soybean (18.25%) compared to teff (81.07%), as soybean’s minimal starch content represents the dominant factor regardless of processing history. The observed trend is therefore attributable to compositional differences rather than processing effects.

These results demonstrate that mixing soybeans into teff flour can substantially improve the macro-nutrients, producing nutrient-dense composite flours suitable for high-protein diets [32]. Overall, blending teff with soybeans significantly enhances the nutritional content while reducing the proportion of carbohydrates. These demonstrate the potential of teff in soybean blends for developing nutrient-dense functional foods, which are required for high-protein and energy-rich diets.

### 2.2. Particle Size Distribution and Viscometric Properties

**Figure 2:**
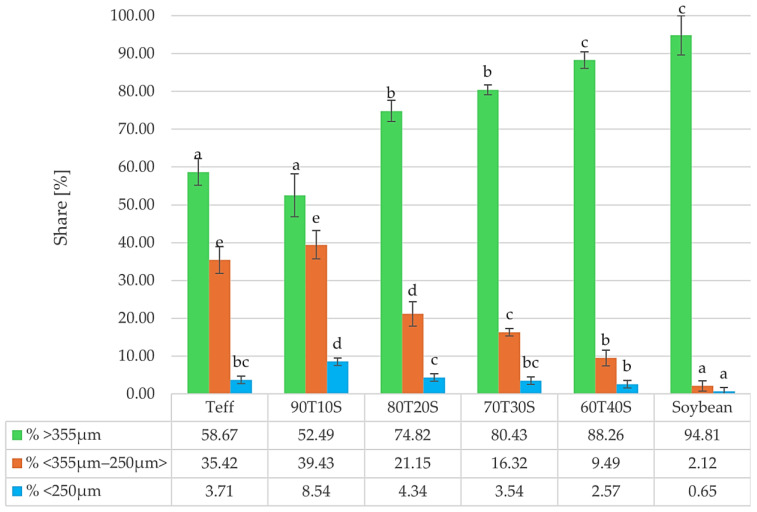
Particle size distribution (%) of flour, teff, soybean, and their blends. T—teff flour, S—soybean flour; sample codes indicate teff/soybean ratio (T:S); e.g., 100T0S = 100% teff, 0% soybean; 60T40S = 60% teff, 40% soybean; different lowercase letters mean significant (*p* = 0.05) differences in size range.

The particle size distributions of teff (T), soybean (S), and their blended flours are shown in Figure 2. Significant differences were observed between the individual flours and the blended flour.

Soybean flour, although visually strongly powdered, behaved as much coarser, with 94.81% of it remaining on the 0.355 mm sieve, while only 2.12% was in the 250–355 μm fraction, and merely 0.65% of particles were finer than 250 μm. The distribution of teff flour granulation (particle size) showed that 58.67% retained on the 0.355 mm sieve, with 35.42% in the 250–355 μm fraction. Additionally, 3.71% of the particles were finer than 250 μm, indicating a relatively finer texture compared to soybean. Such a finer flour form is desirable for baked products, as it facilitates uniform hydration, enhances dough handling, and yields a smoother crumb structure [33]. According to Cauvain [34] and Lin et al. [35], relatively large granules, such as those in soybean flour, may negatively impact dough development and gas retention, ultimately reducing loaf volume and texture quality.

The particle size distribution of teff–soybean blends exhibited intermediate values depending on the inclusion level. For 90T10S, 52.49% of particles were retained on the 0.355 mm sieve, 39.43% in the 250–355 μm fraction, and 8.54% finer than 250 μm. However, as the level of soybean inclusion increases, the distribution progressively shifts toward coarser fractions (74.82% for 80T20S, 80.43% for 70T30S, and 88.26% for 60T40S retained on the 0.355 mm sieve), indicating that a higher soybean inclusion noticeably reduces the flour fineness.

Traditional teff-based applications such as injera (fermented flatbread) and porridges require specific rheological properties for batter formation and paste consistency, which are predominantly contributed by teff’s starch gelatinization characteristics rather than soybean’s protein matrix. Preliminary observations indicate that a soybean inclusion beyond approximately 40% significantly compromises the viscosity and water-holding properties essential for these traditional applications, establishing a practical upper limit for solid food formulation that has not been systematically investigated in relation to nutritional and antioxidant outcomes. Table 2 shows viscometric data of sample flour teff, soybean, and their blends.

Teff flour revealed the highest peak viscosity (12,198 mPa·s), while the soybean flour sample exhibited the lowest value (257 mPa·s) compared to teff, which confirms that soybeans’ limited starch content, protein characteristics, and mainly lipid presence strongly suppress starch gelatinization [36,37]. The non-detectable pasting peak temperature in soy samples further supports its low gelatinization potential [38]. Blends of teff with soybean at different proportions showed intermediate pasting properties. As the level of soybean substitution progressively increased, viscosity decreased. Even at higher soy levels, the blends exhibited a noticeable viscosity, indicating that teff starch still contributed to the pasting properties. The reason limiting or delaying their swelling and pasting capacity could be due to factors like both soybean meal preprocessing (necessary to inactivate phytase, but involves boiling intact grains for 30 min), which can partially gelatinize the residual starch in grain and denaturize proteins, hindering their swelling capacity, and fat content, which can interact with starch by forming a protective layer around granules, limiting their access to water and thus preventing or delaying their swelling and pasting [39,40].

In blended flours, the pasting temperature (PT) ranged from 68.33 to 71.00 °C; however, the pasting temperature of soybean was non-detectable. This is due to the specific content being very low in starch but rich in proteins and lipids. The low starch content associated with proteins and fibers and the high lipid concentration limit water absorption and prevent the swelling and gelatinization of the teff flour [41,42]. As a result, no starch pasting peak was detected, unlike cereals such as teff; instead, heating mainly caused further protein denaturation [43]. On the other hand, peak time (PT) was slightly shorter in blends, likely due to altered water absorption and restricted granule swelling.

The significant viscosity reduction at 10% soybean inclusion (12,198–644 mPa·s, 95% decrease) suggests strong fat–starch interactions. This fat coating effect explains the disproportionate viscosity loss at minimal soybean levels—once the critical coverage threshold is reached, gelatinization is substantially impaired; the lipid content increases from 2.14% (teff) to 4.22% (90T10S). Another factor to consider could be starch dilution in blends with soybean meal addition; however, the very high values for pasting parameters obtained for 100% teff suggest that not only starch dilution is taking place, as the viscosity drops too intensely. A comprehensive investigation of the functional, rheological, and textural properties of these same flour blends, including a detailed analysis of starch dilution effects, particle size correlations, water competition mechanisms, and viscoelastic behaviors under different solvent systems, is provided in our companion study [44].

### 2.3. Color Profile of Composite Flours

The color profiles of teff, soybean, and blends of flours are shown in Table 3.

There was a significant difference (*p* < 0.05) in color values between the sample flours. Blends of teff–soybean flours’ lightness (L*) decreased as the teff proportion increased, with the brightest in soybean flour (93.88) and the darkest in teff (65.78). The change in lightness in the blend flours was attributed to the presence of natural pigments and phenolics in red teff [28,45]. This finding also aligns with that of other researchers, such as Cappa et al. [46], Cordero-Clavijo and Lazo-Vélez [47], and Serna-Saldivar [48], who reported that blending light-colored legumes with pigmented cereals reduces the overall brightness of the flour. The values of redness to greenness (a*) showed that redness was increased with teff inclusion, shifting from negative (greenish) in soy flour (−1.95) to positive (3.10) in teff-rich blends. The change in color from green to red in the blend flours could be due to the consistency with teff, which contains natural reddish-brown pigments [28,49]. The highest yellowness (b*) was observed in soybean flour (24.21), but was lower in teff and the blends, ranging from 13.14 to 14.38, although C was also higher in soy, indicating a brighter color. Soybean flour (30.88) exhibited the largest difference, reflecting a clear contrast with teff, while the blends at 30% and 40% soy showed moderate values of 8.83 and 8.71, respectively, suggesting acceptable visual changes [50]. Generally, soybeans contribute lightness and yellowness, while teff darkens and reddens the blends. Flour blends showed a gradual shift in their color as the formulation level changed. Therefore, a moderate substitution from 20% to 40% soybean balances nutritional benefits with acceptable color quality for food applications [51].

The color parameters exhibit inverse relationships with the antioxidant capacity (Table 3). Brown teff’s elevated redness (a* = 4.06) and reduced lightness (L* = 65.78) correlate with a lower TPC (1.89 mg GAE/g DM), reflecting insoluble bound phenolics in pigmented seed coats that resist extraction yet contribute visual darkness. Conversely, soybean’s higher lightness (L* = 93.88) paradoxically corresponds to a superior TPC (5.77 mg GAE/g DM) and FRAP (7.65 mg/g DM), attributed to colorless isoflavones (genistein, daidzein) dominating its phenolic profile. The correlation analysis reveals a negative association between a* values and extractable polyphenols (r = −0.89, *p* < 0.01), indicating that pigmentation intensity does not accurately predict antioxidant capacity. Color–bioactivity relationships demonstrate the phytochemical diversity between anthocyanin-rich cereals and isoflavone-dominant legumes.

### 2.4. Antioxidant Properties

The total polyphenol content (TPC) and reduced capacity (FRAP) are shown in Table 4.

Pure soybean flour demonstrated a significantly higher total phenolic content (TPC 5.77 mg GAE/g DM) and ferric reducing antioxidant power (FRAP 7.65 mg FeSO_4_/g DM) compared to teff (TPC 1.89 mg GAE/g DM; FRAP 3.68 mg FeSO_4_/g DM), consistent with its richness in isoflavones and bioactive proteins [52,53]. Transition metals (Fe, Cu, Cd) can catalyze pro-oxidant reactions or interfere with ferric reduction assays; their absence ensures that the observed antioxidant capacity genuinely reflects phenolic compounds rather than metal-mediated electron transfer artefacts.

**Figure 3:**
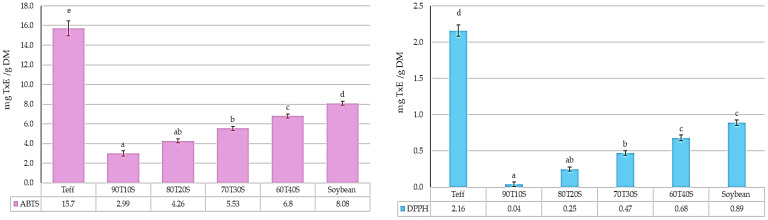
Antioxidant capacity of teff and soybean flours and their blends. Sample codes indicate teff/soybean ratio (T:S); e.g., 100T0S = 100% teff, 0% soybean; 60T40S = 60% teff, 40% soybean; TxE—Trolox equivalents; DM—dry matter. Different lowercase letters indicate significant differences (*p* = 0.05) between the samples.

Meanwhile, pure teff flour exhibited the highest radical scavenging capacity, with DPPH (2.16 mg TxE/g DM) and ABTS (15.7 mg TxE/g DM) values substantially exceeding those of pure soybean flour (DPPH 0.89 mg TxE/g DM; ABTS 8.08 mg TxE/g DM) (Figure 3). This assay-dependent difference reflects the distinct phenolic profiles of these flours: teff’s extractable phenolics, predominantly flavones and phenolic acids [28,54], exhibit a high specific radical scavenging efficiency in DPPH and ABTS assays despite their lower absolute concentration. 

Blending teff with soybean produced predictable compositional effects on TPC and FRAP, which increased proportionally with soybean inclusion. The 60T40S formulation achieved a TPC of 3.44 mg GAE/g DM (82% increase from pure teff) and FRAP of 5.19 mg FeSO_4_/g DM (41% increase). These improvements align with Singh et al. [53], who reported 35–65% TPC increases in cereal–legume blends. Similar cereal–legume systems—wheat–soybean (TPC 2.1–3.8 mg GAE/g) and maize–cowpea (DPPH 0.6–1.2 mg TxE/g) [52]—show comparable antioxidant ranges, confirming that teff–soybean composites achieve competitive bioactive profiles. However, the DPPH and ABTS values in blends were substantially lower than pure teff across all inclusion levels, with 90T10S showing the lowest values (DPPH 0.04 mg TxE/g DM; ABTS 2.99 mg TxE/g DM), progressively increasing with higher soybean ratios (60T40S: DPPH 0.68 mg TxE/g DM; ABTS 6.8 mg TxE/g DM). 

This non-additive pattern suggests that the protein–fiber matrix in composite flours limits phenolic extractability [55,56], or that interactions between teff phenolics and soybean proteins reduce the radical scavenging efficiency in hydroalcoholic extracts. The protein–fiber matrix effect also explains why antioxidant increases (82% TPC) remain proportionally lower than protein enrichment (132% at 60T40S).

Also, Multescu et al. [57] and Do Nascimento et al. [58] confirmed that incorporating legumes into cereal-based flours enhances their phenolic content and antioxidant properties. Similar studies have shown that cereals, such as wheat, maize, millet, and acha, when combined with legumes (soybean, cowpea, and peanut blends [51]), enhance antioxidant activity. 

Soybean’s superior TPC and FRAP capacity primarily derives from isoflavones—genistein, daidzein, and glycitein—which collectively constitute 0.1–0.4% of its dry weight [59]. These polyphenolic compounds exhibit a potent electron-donating capacity through multiple hydroxyl groups on their benzopyrone structure, with genistein demonstrating a particularly high performance in FRAP assays. Additionally, soybeans contain phenolic acids (caffeic, ferulic, sinapic) and condensed tannins that synergistically enhance antioxidant activity [59]. Unlike teff’s predominantly insoluble bound phenolics resistant to extraction [60], soybean isoflavones exist primarily as soluble glycosides (genistin, daidzin) readily extractable under hydroalcoholic conditions [61], explaining soybean’s superior TPC and FRAP performance.

### 2.5. Mid-Infrared Absorption Profile

**Figure 4:**
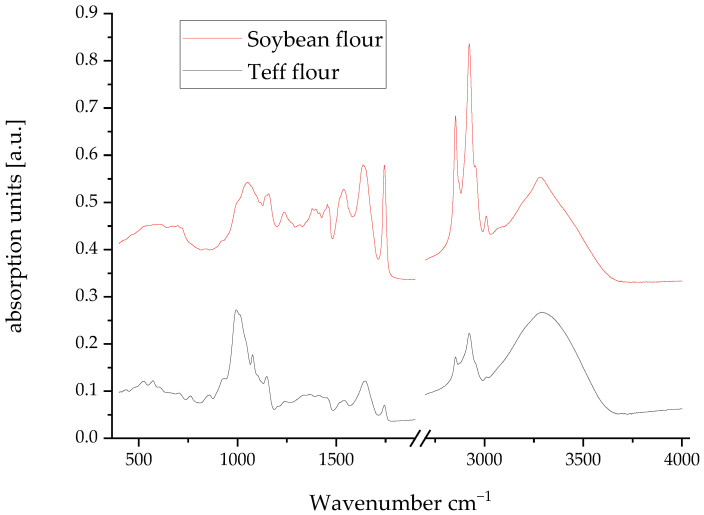
Mid-infrared spectra of teff and soybean flours.

The most informative region of the spectrum is between 800 and 1800 cm^−1^, often referred to as the fingerprint region. In the soybean spectrum, a very strong and broad band is observed, centered around 1650 cm^−1^ and 1540 cm^−1^, which are the characteristic amide I and amide II bands of proteins. The amide I band (primarily C=O stretching) is represented by a peak at 1650.63 cm^−1^ with a substantial area, while the amide II band (N-H bending and C-N stretching) contributes to the complexity in the 1550–1500 cm^−1^ range. This is a signature of the high protein content in soybeans. Furthermore, a distinct peak at 1744 cm^−1^, attributable to the C=O stretching of esters, likely from lipid components, is present and notable for its high maximum height, confirming soybean’s significant fat content. The region between 1200 and 900 cm^−1^ exhibits multiple peaks, such as those at 1157 cm^−1^ and 1050 cm^−1^, which are associated with the C-O stretches and C-C vibrations of carbohydrates, although they are less dominant compared to the protein peaks. The spectrograms of both flours, teff and soybean, are shown in Figure 4.

In contrast, teff flour presents a markedly different spectral profile. Protein-associated amide I and II bands appear less intense relative to soybean. The dominant feature is the complex absorption pattern in the 1200–900 cm^−1^ region, characteristic of carbohydrate fingerprints. Specifically, the peak at ~1150 cm^−1^ corresponds to the C-O-C asymmetric stretching of glycosidic linkages in starch. The strong absorption at 1087 cm^−1^ represents C-O stretching vibrations in the C-O-H groups of glucose units. The peaks at 1009 and 999 cm^−1^ indicate C-O and C-C stretching coupled with ring vibrations in the pyranose structures of amylose and amylopectin. The broad absorption at 1047 cm^−1^ reflects ordered crystalline regions within starch granules. This spectral signature confirms teff’s starch-dominated composition (81% carbohydrates), with the lipid ester carbonyl peak (~1740 cm^−1^) appearing weaker than that of soybean, consistent with its lower fat content (2.14%).

Soybean flour is a well-established source of high-quality plant protein and lipids, which is confirmed by its strong amide and ester carbonyl peaks. However, its carbohydrate profile is less complex and is often lower in certain beneficial fibers. Teff flour, on the other hand, is spectrally defined by its rich and complex carbohydrate matrix. Therefore, enriching soybeans with teff is a highly valuable nutritional strategy. This combination creates a composite ingredient that is not only protein- and lipid-rich but also fiber-rich. The proteins and fats from soybeans provide essential amino acids and energy, while the carbohydrates from teff ensure a slower, more sustained release of glucose, supporting a healthy gut microbiome. From a functional perspective, teff’s starch can also improve the texture and water-binding capacity of food products, mitigating the sometimes dense or dry texture associated with high-soybean formulations. 

### 2.6. Principal Component Analysis

**Figure 5:**
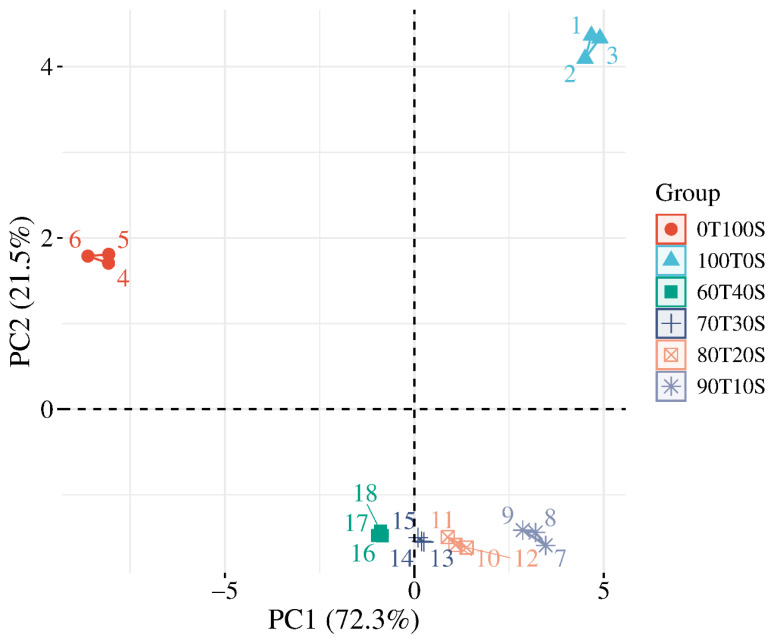
Factor map of the PCA performed on 18 observations and 25 variables. Six cluster groups were identified, corresponding to different samples.

The principal component analysis of 25 physicochemical parameters effectively differentiated teff and soybean flour blends (Figure 5). The first two principal components explained 91.6% of the total variance, with PC1 accounting for 70.6% and PC2 contributing 21.0%, demonstrating a strong dimensionality reduction and clear ingredient-based separation. PC1 functions as the primary compositional gradient reflecting the inverse relationship between starch-rich teff and protein-rich soybean characteristics, while PC2 captures variations in water-mediated functional properties, particularly the pasting behavior during thermal processing.

Teff samples, 100T0S, occupy the extreme positive PC1 region, with scores ranging from +4.50 to +4.90, combined with positive PC2 scores from +4.09 to +4.36. This positioning reflects the high carbohydrate content, strong pasting properties from starch gelatinization, and lower protein and antioxidant density characteristics of teff flour. Pure soybean samples, 0T100S, cluster in the extreme negative PC1 region, with scores from −8.07 to −8.61 alongside positive PC2 values from +1.71 to +1.81. The strong negative PC1 positioning confirms elevated protein, fat, fiber, and antioxidant content, along with a minimal carbohydrate concentration and substantially reduced pasting capacity in soybean flour.

Blend samples demonstrate a systematic progression along PC1 corresponding to formulation ratios. The 90T10S formulation with minimal soybean substitution occupies the moderately positive PC1 space from +2.86 to +3.46, with strongly negative PC2 scores from −1.41 to −1.59, maintaining a largely teff-like composition while exhibiting a distinctly altered pasting behavior. Progressive soybean incorporation shifts samples toward negative PC1 values as protein and fat contents increase, while the carbohydrate concentration decreases. Intermediate formulations 80T20S and 70T30S position near PC1 values from +0.88 to +1.37 with negative PC2, displaying transitional properties between the pure ingredient extremes. The 60T40S blend achieves the most balanced intermediate positioning, from +0.10 to +0.25 on PC1, while maintaining a negative PC2, indicating a substantial nutritional enhancement through soybean fortification with a continued modulation of functional characteristics.

The substantial 21.5% variance captured by PC2 reveals that pasting and hydration properties vary independently of basic macronutrient compositions. This independence suggests that functional properties are influenced by factors beyond simple protein-to-carbohydrate ratios, likely involving specific starch structural characteristics, protein functionality, lipid interactions, and particle surface properties. The positioning of blend samples indicates that pasting properties do not follow the linear compositional gradient observed for nutritional parameters. Blends exhibit systematically diminished pasting characteristics compared to pure teff despite intermediate macronutrient profiles, suggesting antagonistic interactions between teff starch and soybean protein–lipid components during thermal processing. This deviation from additive behavior necessitates empirical testing for achieving target pasting properties rather than relying on a simple interpolation between pure ingredient values, with important implications for formulation optimization targeting specific processing requirements alongside nutritional objectives. The observed independence between nutritional composition (PC1: 72.3%) and pasting properties (PC2: 21.5%) reflects distinct molecular mechanisms governing each parameter. The nutritional composition follows a predictable mass–balance dilution—protein, fat, and fiber linearly interpolate between teff and soybean endpoints based on blend ratios. 

However, the pasting behavior depends on starch granule hydration kinetics, where even minimal soybean protein (13.21% at 90T10S) forms amphiphilic interfacial layers on granule surfaces through hydrophobic interactions between protein β-sheets and amylose helices. These protein films create diffusional barriers that reduce water influx rates, disproportionately suppressing gelatinization relative to compositional changes. Additionally, soybean lipids form amylose–lipid inclusion complexes (evidenced by FT-IR ester peaks), further restricting granule swelling. This non-linear, threshold-dependent behavior operates independently of simple nutrient dilution effects.

## 3. Materials and Methods

### 3.1. Raw Materials and Blend Preparation and Characterization

The experiment was conducted at the Department of Biotechnology and Food Analysis, Adaptive Food Systems Accelerator—Science Centre, Wrocław, Poland. Teff (*Eragrostis tef* (Zucc.) Trot.) var. Quyi grains were purchased from the local market in Gondar City, Ethiopia, and soybeans (*Glycine max* (L.) Merr.); var. Afgat were collected from the Gondar Agricultural Research Centre, Gondar, Ethiopia.

Teff was harvested from mid-November to December and left in the field for several days to dry. It was then threshed, cleaned, and dried (12% moisture) before storage. After drying, it was stored in a traditional structure called a Gotera/Gota granary made of local materials like wood, mud, and teff straw. Soybeans were carefully dried to a moisture content of less than 13% for safe storage after being collected at the proper moisture level to prevent shattering. For better ventilation and to avoid contact with ground moisture or pests, the dry-harvested grains were stored in clean sacks on a wooden pallet or elevated stand away from walls. Finally, these were stored in a cold, dark, and well-ventilated area.

The soybeans were cleaned, boiled (for 30 min), dehulled, dried at 60 °C for about 13 h, and milled into flour. All flours were packed in polyethylene plastic bags, labelled, and stored at 4 °C until analysis to prevent oxidative degradation.

For blends, a rotary electric mixer KM 3400 (CLATRONIC sp z o.o., Opole, Poland) was used. The 0–40% soybean inclusion range was selected based on preliminary observations indicating that soybean concentrations exceeding 40% substantially impaired the consistency required for dough, thereby establishing a practical upper limit for formulation. Flour blends of teff and soybean were prepared at ratios of 90%, 80%, 70%, and 60% (teff flour—T) and 10%, 20%, 30%, and 40% (soybean flour—S), respectively. The blends were mixed for 10 min at room temperature. The blends were coded based on their composite ratios as 100T0S, 0T100S, 90T10S, 80T20S, 70T30S, and 60T40S. Finally, each blended flour was packed in Ziploc polyethene bags, labelled, and stored for further analysis.

### 3.2. Granulometric Analysis

Granulometric analysis of each sample flour and the blends was determined using the AACC 66-20.01 method [62]. The samples were sieved (50 g) using a vibratory sieve shaker LPzE-2e (Multiserw Morek, Brzeźnica, Poland) at 0.65 mm vibration amplitude for 10 min with screens of 80, 106, 125, 150, 180, and 355 μm. The percentage of each particle size fraction was reported only as >355 μm, 250–355 μm, and <150 μm, as the weight of specific fractions was negligible. Each sample of flour was analyzed in triplicate.

### 3.3. Pasting Properties: Viscoamylographic Tests (RVA)

The pasting characteristics of the sample flours and their blends were determined according to AACC (2010) using the Rapid Visco Analyser Starch Master2 (Newport Scientific, Sydney, Australia) [63,64,65]. A standard amount of sample and solvents was adjusted using the ThermoCline v. 3.17.5.15. Distilled water was used as a solvent to see the pasting characteristics of individual sample flours and blends. The temperature profile implemented in all the blends followed these parameters: initial temperature at 50 °C for 2 min; heating from 50 °C to 95 °C for 4 min; then holding for 5 min; and finally cooling for 4 min to 50 °C. The peak viscosity (PV), trough viscosity (TV), breakdown viscosity (BV), final viscosity (FV), setback viscosity (SV), peak time (min), and pasting temperature (°C) were measured and calculated. The analysis for each sample was carried out in triplicate.

### 3.4. Proximate Composition

The ash content of flour samples and blends was determined using the AOAC method (AOAC, 2016) [66]. A total of 3 g of sample was placed in a pre-weighed crucible. The samples were heated before muffle furnace incineration. Then, the samples were burned in a muffle furnace at 550 °C for 5 h. The dry ash weight percentage was calculated according to Equation (1):

(1) $$Ash\left(\%\right)=\frac{\left(a\;-\;b\right)*\;10000}{\left(x\;-\;b\right)*\left(100\;-\;y\right)}$$

where a = mass of crucible with ash (g); b = mass of empty crucible (g); x = mass of crucible with sample before ashing (g); y = moisture content of sample (%).

The nitrogen content of each flour and blend was analyzed using the Kjeldahl method, and protein content was estimated using a nitrogen-to-protein conversion factor of 6.25 (AOAC method 954.01) [67]. A total of 0.5 g of ground sample was added to 20 cm^3^ of concentrated sulfuric acid and 2 Kjeldahl tablets (containing 3.5 g K_2_SO_4_ and 0.4 g CuSO_4_·5H_2_O) for sample mineralization. Distillation was performed using KjelFlex K-360 (BÜCHI Labortechnik GmbH, Essen, Germany) following the manufacturer’s procedure. Titration was conducted by adding approximately 5 drops of Toshiro indicator to the conical flask placed in the distiller, followed by titration with 0.1 M HCl until the color changed or pH reached approximately 4.6. The nitrogen content in the sample N (%) and protein content Y (%) were calculated according to Equations (2) and (3):

(2) N=((a+b)∗n∗M(N)∗F)/(m∗1000)∗100%

(3) %Protein=%N×6.25 (teff) 5.71 (soy)

where a = volume of standard HCl used for sample titration (cm^3^); b = volume of standard HCl used for titration of the blank sample (cm^3^); n—molarity of HCl; M(N) = atomic mass of nitrogen (14.007 g/mol); F = molar reaction coefficient (for HCl = 1); m = mass of the tested sample (g); 1000-conversion factor (change unit from cm^3^ to dm^3^).

Crude fat Soxhlet extraction was carried out with the Soxhlet Extractor (SOX606, Guangzhou, China) in accordance with the apparatus manufacturer’s procedure and AOAC methods (method 920.39) (AOAC, 2005) [68]. A total of 5 g of flour sample was extracted using 100 mL of petroleum ether. Extraction was conducted at a temperature of 70 °C, a time of 90 min, and a pre-drying time of 20 min. After the extraction of fat was carried out, the sample containing thimbles was allowed to be oven-dried at 50 °C for 20 min and then at 100 °C for 1 h. The samples were then allowed to cool to room temperature until they reached a constant weight. Extraction experiments were performed in triplicate. The fat content was calculated as in Equation (4):

(4) Crude Fat (%)=(M1−M0)/M × 100% .

where M1—mass of solvent cup and crude fat (g); M0—mass of drying solvent cup (g); M—mass of sample (for the sample after the moisture analysis, calculated according to the mass before the moisture analysis) (g).

Total dietary fiber (TDF) was determined using the Megazyme K-TDFR-100A/K-TDFR-200A 04/17 (Megazyme, Wicklow, Ireland) kit following AOAC 991.43 [69]. Samples (1 g) were enzymatically digested (α-amylase, protease, amyloglucosidase), precipitated with ethanol, filtered, and dried (105 °C). Crucibles were ashed (525 °C, 5 h), and TDF (%) was calculated gravimetrically [69]. All analyses were performed in triplicate with blank corrections. Finally, samples of dietary fiber analysis were calculated as Equation (5).

Dietary Fiber (%) = ((R_1_ + R_2_)/2 − P − A − B)/((m_1_ + m_2_)/2) × 100 (5)

$$B=\frac{BR1+BR2}{2}-BP-BA$$

where R_1_ = residue weight 1 from m_1_ in mg; R_2_ = residue weight 2 from m_2_ in mg; m_1_ = sample weight 1 in g; m_2_ = sample weight 2 in g; A = ash weight from R_1_; P = protein weight from R_2_; B = Blank.

Heavy metals (cadmium, arsenic, lead, mercury) were determined using Inductively Coupled Plasma Mass Spectrometry Agilent 7900 (Agilent Scientific Instruments, Santa Clara, CA, USA) following microwave-assisted acid digestion (HNO_3_/H_2_O_2_). Samples (0.5 g) were digested in 8 mL concentrated HNO_3_ and 2 mL H_2_O_2_ at 200 °C for 20 min. Calibration was performed using certified reference standards (Merck KGaA, Darmstadt, Germany). Detection limits were Cd (0.002 mg/kg), As (0.010 mg/kg), Pb (0.005 mg/kg), and Hg (0.001 mg/kg). All analyses were performed in triplicate.

### 3.5. Color Analysis

The color of all flour samples was measured using a colorimeter (model CR-310, Konica Minolta Sensing Inc., Tokyo, Japan), which had been previously calibrated against a standard white tile. The CR-A50 attachment for granular sample measurement was used to measure the blend’s color. The CIELab color system consists of a luminance or lightness component (L*) and two chromatic components: the (a*) component for green (−a) to red (+a) and the (b*) component from blue (−b) to yellow (+b) colors. Each sample of flour and its blends was measured in triplicate to ensure accuracy, and the average value was then determined for final analysis. The chroma (C) was calculated according to Equation (6).

(6) $$C=\sqrt{{\left(a\right)}^{2}+{\left(b\right)}^{2}}$$

### 3.6. Antioxidant Analysis of Flours and Blends

Bioactive compounds were extracted using two solvent systems, distilled water and a hydroalcoholic mixture (ethanol/water, 80:20 *v*/*v*), each acidified with 1% HCl. The alcohol-to-water ratio used (80:20 *v*/*v*) was optimal in terms of extraction effectiveness and efficiency. The 1% addition of hydrochloric acid facilitated the decomposition of cell walls of the raw material. Exactly 0.5 g of the dried flour sample was weighed and suspended in 10 mL of the extraction solvent. The mixtures were vortexed for 1 min (MX-S, Chemland, Stargard, Poland) and agitated for 2 h at ambient temperature using a laboratory rotary shaker (MX-RD PRO, Chemland, Stargard, Poland). After incubation, samples were centrifuged at 3500× *g* for 10 min at 4 °C (MPW-350, MPW MED. INSTRUMENTS, Warsaw, Poland). The obtained supernatants were stored at 8 °C and analyzed within 24 h.

An antioxidant analysis of each flour sample and blended flours was performed using spectrophotometric analysis. The extraction was conducted using a laboratory scale rotary shaker. The supernatant extract was collected for the determination of total phenolic content (TPC), antioxidant activity via ferric reducing antioxidant power (FRAP assay), 2,2-diphenyl-1-picrylhydrazyl radical (DPPH assay), diammonium 2,2′-azinobis [3-ethyl-2,3-dihydrobenzothiazole-6-sulphonate] (ABTS assay), and reducing sugar content.

The reducing sugar content in the extracts was determined using a modified dinitrosalicylic acid (DNS) colorimetric method [70]. A 0.5 mL aliquot of each extract was mixed with 0.25 mL of DNS reagent and heated in a boiling water bath for 5 min. After cooling to 50–60 °C, 3 mL of distilled water was added to dilute the reaction mixture. The absorbance was measured at 530 nm using a UV-Vis spectrophotometer (VIS V5100, Metash Instruments, Shangai, China). Glucose standards ranging from 100 to 800 µg/mL were used for calibration. The absorbance of a blank sample containing 0.5 mL of distilled water instead of the test extract was also measured. Results were expressed as milligrams of glucose equivalents (GluE) per gram of dry sample (GluE mg/g DM).

The total phenolic content was determined using the Folin–Ciocalteu colorimetric method. Briefly, 20 μL of extract was combined with 1580 μL of distilled water, followed by the addition of 100 μL of undiluted Folin–Ciocalteu reagent. After vortexing and a 6 min incubation at room temperature (25 °C), 300 μL of saturated CaCO_3_ solution was introduced and mixed until stable blue coloration developed. Samples were incubated in darkness at 38 °C for 30 min using a water bath (06-DK-98-IV, ChemLand, Stargard, Poland), and absorbance was recorded at 765 nm (VIS V5100, Metash Instruments, Shangai, China). Analyses were performed in triplicate, with results expressed as milligrams of gallic acid equivalent (GAE) per gram of dry matter (DM) [71]. 

Antioxidant activity was assessed using the DPPH free radical scavenging assay [72]. For each measurement, 0.0345 mL of the extract was added to 1 mL of a 0.1 mM methanolic DPPH solution. The mixture was incubated for 20 min in the dark at room temperature, and absorbance was measured at 517 nm (VIS V5100, Metash Instruments, Shangai, China). The absorbance of a blank sample was also measured, which contained 1 mL of DPPH working solution and 0.0345 mL of distilled water instead of the tested extract. A calibration curve was prepared using Trolox as the standard (100–800 mg/L). The results were expressed as milligrams of Trolox equivalents (TxE) per gram of dry sample (mg TxE/g DM).

The ABTS radical scavenging capacity was evaluated using a previously described method [73], with minor modifications [74]. Briefly, 0.0204 mL of extract was added to 1 mL of a freshly prepared ABTS^•+^ solution, and the absorbance was measured at 734 nm (VIS V5100, Metash Instruments, Shangai, China) precisely 10 s after mixing. The absorbance of a blank sample was measured, which contained 1 mL of ABTS working solution and 0.0204 mL of distilled water instead of the tested extract. Trolox was used as a standard (100–800 mg/L), and the antioxidant capacity was reported as mg TxE/g DM.

The FRAP assay was used to determine the electron-donating ability of the flour extracts [75]. A 0.0345 mL aliquot of each extract was mixed with 0.998 mL of freshly prepared FRAP reagent. After incubation for 15 min at 36 °C, absorbance was measured at 593 nm (VIS V5100, Metash Instruments, Shangai, China). The absorbance of a blank sample was measured, which contained 0.998 mL of FRAP working solution and 0.0345 mL of distilled water instead of the test extract. The results were calculated using a ferrous sulfate standard curve (100–800 µmol/L) and expressed as milligrams of FeSO_4_ equivalents per gram of dry sample (mg FeSO_4_/g DM).

### 3.7. Mid-Infrared (FT-IR/ATR) Spectroscopy

The dried flour samples were used directly for FT-IR analysis. FT-IR spectra were acquired using a Nicolet 6700 spectrophotometer (Thermo Fisher Scientific, Waltham, MA, USA) equipped with a diamond crystal in attenuated total reflectance (ATR) mode. Each sample was scanned 128 times in the mid-infrared region (4000–500 cm^−1^) at a resolution of 2 cm^−1^. The wavenumbers and corresponding band assignments for all samples were recorded. All measurements were performed in triplicate. Spectral data were processed and compared using OriginPro 2024b software (OriginLab Corporation, Northampton, MA, USA).

### 3.8. Statistical Analysis

All results are expressed as mean values accompanied by standard deviations (±SD). The variance of the results (ANOVA) and PCA were evaluated using Statgraphics Centurion software (v. XVIII, StatPoint Technologies, Inc., Warrenton, VA, USA). ANOVA was performed with a previous normality check of the data using a *p*-value < 0.05 significance level. Differences between group means were assessed for statistical significance at a confidence level of *p* ≤ 0.05 using Tukey’s Multiple Range Test. A PCA test was performed on 6 samples and 25 parameters. A bootstrap graph was generated on 18 samples—6 original samples with 2 replicates each—to assess repeatability. Bootstrap graphs were prepared using the SRPlot platform [76].

## 4. Conclusions

The principal component analysis revealed that nutritional composition and pasting properties vary independently, requiring empirical optimization rather than simple interpolation. Soybean proteins and lipids interfere with teff starch gelatinization, causing dramatic viscosity reductions that establish practical formulation boundaries for paste-forming applications.

Traditional paste-forming products, such as injera and porridges, benefit from blends containing 20–30% soybean, which maintain an adequate viscosity while achieving amino acid complementation and enhancing antioxidant capacity. High-protein functional foods where viscosity is non-critical can utilize formulations with 30–40% soybean inclusion to maximize the nutritional density while preserving bioactive compound profiles.

This research systematically characterizes compositional–functional relationships across the complete formulation space, demonstrating that nutritional and processing objectives operate as independent optimization axes. Future investigations should evaluate processing interventions including extrusion, fermentation, and enzymatic modification to address functional limitations. Complementary studies on sensory characteristics, consumer acceptance, and in vivo bioavailability would facilitate commercial translation. Exploring ternary blends incorporating additional nutrient-dense ingredients could further enhance these naturally gluten-free composite systems.

## Figures and Tables

**Table 1:** The proximate composition and heavy metal content of teff and soybean flours and their blends.

Samples	Ash		Protein		Fat		Fiber		CoH		Energy (Kcal)	
	[% DM]											
0T100S	4.38 ± 0.06 ^f^		42.77 ± 0.33 ^f^		22.95 ± 0.38 ^f^		11.65 ± 0.05 ^f^		18.25± 0.72 ^f^		450.62 ± 1.88 ^f^	
100T0S	3.43 ± 0.02 ^e^		9.93 ± 0.54 ^a^		2.14 ± 0.17 ^a^		3.43 ± 0.02 ^a^		81.07 ± 0.34 ^a^		383.32 ± 0.67 ^a^	
90T10S	2.72 ± 0.02 ^a^		13.21 ± 0.48 ^b^		4.22 ± 0.16 ^b^		4.25 ± 0.02 ^b^		75.60 ± 0.32 ^b^		393.22 ± 0.76 ^b^	
80T20S	2.90 ± 0.03 ^b^		16.5 ± 0.43 ^c^		6.3 ± 0.16 ^c^		5.07 ± 0.01 ^c^		69.23 ± 0.29 ^c^		399.62 ± 0.78 ^c^	
70T30S	3.09 ± 0.03 ^c^		19.79 ± 0.39 ^d^		8.39 ± 0.18 ^d^		5.89 ± 0.01 ^d^		62.84 ± 0.29 ^d^		406.03 ± 0.84 ^d^	
60T40S	3.27 ± 0.04 ^d^		23.07 ± 0.35 ^e^		10.47 ± 0.2 ^e^		6.72 ± 0.02 ^e^		56.47 ± 0.32 ^e^		412.39 ± 0.94 ^e^	
**Heavy metals [mg/kg DM]**												
	Cd			As			Pb			Hg		
0T100S	0.0039 ± 0.0006			<0.010 ± 0.002			0.022 ± 0.003			<0.0010 ± 0.0002		
100T0S	<0.0020 ± 0.0003			<0.010 ± 0.002			0.105 ± 0.016			<0.0010 ± 0.0002		

T—teff flour, S—soybean flour; sample codes indicate teff/soybean ratio (T:S); e.g., 100T0S = 100% teff, 0% soybean; 60T40S = 60% teff, 40% soybean; DM—dry matter; CoH—carbohydrates; different lowercase superscript letters in the column indicate significant (*p* = 0.05) differences among the values.

**Table 2:** Viscometric characteristics of teff and soybean flours and their blends.

Samples	PV (mPa·s)	TV (mPa·s)	BV (mPa·s)	FV (mPa·s)	SBV (mPa·s)	PT (s)	PTemp. (°C)
0T100S	257 ± 25 ^bc^	116 ± 90 ^a^	142 ± 16 ^bc^	611 ± 41 ^c^	496 ± 32 ^d^	7.0 ± 0 ^d^	n.d.
100T0S	12,198 ± 133 ^e^	2473 ± 23 ^e^	9725 ± 13 ^d^	6611 ± 36 ^e^	4137 ± 33 ^f^	7.5 ± 0.31 ^e^	68.9 ± 0.3 ^b^
90T10S	644 ± 60 ^d^	455 ± 40 ^d^	190 ± 30 ^c^	1037 ± 11 ^d^	583 ± 70 ^e^	5.31 ± 0.03 ^c^	71.00± 0 ^c^
80T20S	331 ± 80 ^c^	263 ± 50 ^c^	68 ± 30 ^ab^	616 ± 14 ^c^	353 ± 80 ^c^	5.00 ± 0.00 ^b^	70.33 ± 0.58 ^c^
70T30S	218 ± 20 ^ab^	190 ± 15 ^b^	28 ± 40 ^a^	447 ± 33 ^b^	256 ± 16 ^b^	4.73 ± 0.06 ^a^	68.33 ± 0.58 ^b^
60T40S	129 ± 90 ^a^	125 ± 70 ^a^	3 ± 30 ^a^	284 ± 13 ^a^	159 ± 90 ^a^	4.67 ± 0.12 ^a^	68.67 ± 1.15 ^b^

T—teff flour; S—soybean flour; sample codes indicate teff/soybean ratio (T:S); e.g., 100T0S = 100% teff, 0% soybean; 60T40S = 60% teff, 40% soybean; PV—pasting viscosity; TV—through viscosity; BV—breakdown viscosity; FV—final viscosity; SBV—setback viscosity; PT—peak time; PTemp—peak temperature; n.d.—non detectable, different lowercase superscript letters mean significant (*p* = 0.05) differences in column.

**Table 3:** The color parameters of teff and soybean flours and their blends.

Samples	L*	a*	b*	C*
0T100S	93.88 ± 1.56 ^e^	−1.95 ± 0.27 ^a^	24.21 ± 0.96 ^b^	24.29 ± 0.93 ^d^
100T0S	65.78 ± 1.36 ^a^	4.06 ± 0.17 ^c^	12.97 ± 0.19 ^a^	13.59 ± 0.22 ^a^
90T10S	68.97 ± 0.98 ^b^	4.27 ± 0.05 ^c^	13.40 ± 0.14 ^a^	14.06 ± 0.15 ^c^
80T20S	80.24 ± 1.34 ^d^	4.36 ± 0.32 ^c^	14.38 ± 1.47 ^a^	15.03 ± 1.43 ^b^
70T30S	74.50 ± 1.28 ^c^	3.51 ± 0.19 ^b^	14.19 ± 0.22 ^a^	14.62 ± 0.25 ^c^
60T40S	74.32 ± 0.30 ^c^	3.10 ± 0.10 ^b^	14.37 ± 0.07 ^a^	14.70 ± 0.10 ^c^

Different lowercase superscript letters mean significant (*p* = 0.05) differences in the column. L* = Lightness; a* = Red–Green; b* = Yellow–Blue; C* = Chroma; T—teff; S—soybean; sample codes indicate teff/soybean ratio (T:S); e.g., 100T0S = 100% teff, 0% soybean; 60T40S = 60% teff, 40% soybean.

**Table 4:** Total polyphenols and ferric reduction capacity of teff and soybean flours and their blends.

Sample	TPC (mg GAE/g DM)	FRAP (mg FeSO_4_/g DM)
0T100S	5.77 ± 0.41 ^d^	7.65 ± 1.30 ^d^
100T0S	1.89 ± 0.09 ^a^	3.68 ± 0.28 ^a^
90T10S	2.28 ± 0.03 ^ab^	4.08 ± 0.12 ^ab^
80T20S	2.67 ± 0.15 ^b^	4.47 ± 0.03 ^b^
70T30S	2.86 ± 0.03 ^bc^	4.82 ± 0.14 ^bc^
60T40S	3.44 ± 0.09 ^c^	5.19 ± 0.35 ^c^

Different lowercase superscript letters mean significant (*p* = 0.05) differences in column; T—teff; S—soybean; sample codes indicate teff/soybean ratio (T:S); e.g., 100T0S = 100% teff, 0% soybean; 60T40S = 60% teff, 40% soybean; GAE—gallic acid equivalent; DM—dry matter.

## Data Availability

The raw data supporting the conclusions of this article are available in the main text of the manuscript.

## Abbreviations

ABTS	2,2′-azino-bis(3-ethylbenzothiazoline-6-sulfonic acid)
BV	Breakdown Viscosity
CoH	Carbohydrates
DM	Dry Matter
DPPH	2,2-diphenyl-1-picrylhydrazyl
FRAP	Ferric Reducing Antioxidant Power
FT-IR	Fourier Transform Infrared Spectroscopy
FV	Final Viscosity
GAE	Gallic Acid Equivalent
GE	Glucose Equivalent
PC	Principal Component
PCA	Principal Component Analysis
PT	Peak Time
PTemp	Peak Temperature
PV	Peak Viscosity
SAA	Sulfur-Containing Amino Acids
SBV	Setback Viscosity
TDF	Total Dietary Fiber
TE	Trolox Equivalent
TPC	Total Phenolic Content
TV	Trough Viscosity
